# The effect of *Abelmoschus esculentus* L. (Okra) extract supplementation on dietary intake, appetite, anthropometric measures, and body composition in patients with diabetic nephropathy

**DOI:** 10.34172/hpp.2022.21

**Published:** 2022-08-20

**Authors:** Omid Nikpayam, Ehsan Safaei, Nazgol Bahreyni, Vahideh Sadra, Maryam Saghafi-Asl, Laleh Fakhr

**Affiliations:** ^1^Student Research Committee, Tabriz University of Medical Sciences, Tabriz, Iran; ^2^Nutrition Research Center, School of Nutrition and Food Sciences, Tabriz University of Medical Sciences, Tabriz, Iran; ^3^Endocrine Research Center, Tabriz University of Medical Sciences, Tabriz, Iran; ^4^Drug Applied Research Center, Tabriz University of Medical Sciences, Tabriz, Iran; ^5^Department of Clinical Nutrition, School of Nutrition and Food Sciences, Tabriz University of Medical Science, Tabriz, Iran

**Keywords:** Abelmoschus, Diabetic nephropathy, Anthropometry, Appetite, Clinical trial

## Abstract

**Background:** Diabetes is a risk factor for chronic kidney disease because it induces nephropathy. Okra is a rich source of antioxidants, vitamins, minerals, and fibers, of which favorable effects in diabetes have been reported in many animal studies. The present trial aimed to investigate the effect of dried okra extract (DOE) supplementation on anthropometric measures, body composition, appetite, and dietary intake in diabetic nephropathy (DN) patients.

**Methods:** In this triple-blind placebo-controlled randomized clinical trial, 64 DN patients were randomly allocated to receive a 125-mg capsule of DOE (n=32) or placebo (n=32) for 10 weeks. At baseline and endpoint of the trial, anthropometric variables, body composition indices, dietary intake, and appetite scores were evaluated.

**Results:** The results showed that energy (*P*=0.047, CI: -425.87, -3.25, ES: 0.539) and carbohydrate (*P*=0.038, CI: -85.64, -2.53, ES: 0.555) intake as well as desire to eat salty food (*P*=0.023) were reduced in DOE group at the endpoint, compared to the baseline values. However, anthropometric measures, body composition, and appetite score were not significantly different between the two study groups.

**Conclusion:** In conclusion, the present clinical trial showed that DOE could significantly decrease energy intake and carbohydrate consumption in the DN patients. Further clinical trials are needed to determine the effects of this supplement.

## Introduction

 Diabetes mellitus (DM) is one of the important public health issues in different societies.^1^ According to the International Diabetes Federation (IDF) reports in 2017, there are 451 million adults with diabetes in the world.^2^ DM is a metabolic disease diagnosed by high blood glucose. In fact, increased blood glucose in diabetic patients impairs the vessels and various organs in the body.^3^ Diabetic nephropathy (DN) is one of the major microvascular complications of diabetes.^4^ DN occurs in nearly 20-30% of diabetic patients.^5^ DN is more common in patients with type 1 diabetes mellitus (T1DM), compared to type 2 diabetic patients. However, due to the higher prevalence of T2DM, the number of DN patients with this type of DM is higher.^6^ DN is characterized by reduced kidney function or glomerular filtration rate (GFR), ^7^ as well as increased creatinine in the serum and urinary albumin excretion.^8^ It is the major cause of end-stage renal disease (ESRD), as well as a financial burden on health system.^9^

 Previous evidence indicated that the storage of high-fat content in the body induces obesity consequently, triggering a low-grade inflammation in the body. It is already reported that the increment of inflammation in the body leads to an increase in blood glucose level and subsequent diabetes. Therefore, body weight (BW) is considered a major risk factor for diabetes.^10^ Also, obesity has a crucial role in the development of DN. In fact, in addition to diabetes, obesity also induces hypertension via increasing renal tubular reabsorption and creating a hypertensive change in renal pressure through several mechanisms, including sympathetic nervous system, renin-angiotensin-aldosterone system, and physical pressure on the kidney. Hypertension leads to increased GFR as well as renal injury induction.^11^

*Abelmoschus esculentus* (okra) is a yearly plant from Malvaceae family, which grows in tropical and subtropical regions.^12^ The main components of okra are dietary fibers, polysaccharides, vitamins, minerals, and antioxidant ingredients such as flavonoids and quercetin.^13^ The favorable effects of okra have been illustrated in various situations such as oxidative stress, inflammation, and tumor process.^14^ Recently, the desirable effects of okra on the management of BW has been cited in several studies.^15,16^ For instance, Dubey and Mishra reported that okra seed supplementation had desirable effects on BW and serum cholesterol level in hypercholesterolemic rats.^17^ Another study on diabetic rats showed that okra powder supplementation could significantly decline BW.^18^ Further, it was shown that antioxidants of okra prevented weight gain without any changes in food intake.^19^ In contrast, Wang et al illustrated that okra powder did not have any significant effect on BW of rats fed a hyperlipidemic diet plus okra powder compared to rats on a hyperlipidemic diet alone.^20^ Also, a clinical trial on diabetic patients did not find any significant effect on BW after 8 weeks of okra powder supplementation.^21^

 Due to the high global prevalence of DN, its huge costs on healthcare system, limited documents about the effects of okra on anthropometric measures in human samples,^21,22^ as well as concern over the relation between BW, hypertension, and kidney function, the present study was conducted to investigate the effects of okra extract supplementation on satiety, body weight, and body composition in DN patients.

## Materials and Methods

###  Preparation of okra extract 

 Preparing dried okra extract (DOE), 150-kg okra fruit was first prepared from Fruit and Vegetable Market of Tabriz in July 2019. The whole okra fruit was washed with water and tinily chopped for better and earlier drying. Then, the chopped okra was placed in the shade at 45°C to dry. Totally, 15 kilograms of okra powder was obtained from fresh okra fruits. After that, the okra powder was mixed with 50% alcohol in a ratio of 1 to 5 and placed in a percolator device at a temperature of 35 degrees centigrade, and the circulation operation was performed for 24 to 48 hours. After saturating the solvent and measuring the concentration of the solution, the mixture was filtered and transferred to a rotary device. Under vacuum conditions and at a temperature of 40 to 45°C, the hydraulic solvent was separated; then, the okra solution was concentrated. Finally, the concentrated okra was transferred to a vacuum dryer device; then, it was dried and pulverized.^23^

###  Participants 

 The present 10-week randomized, triple-blinded, placebo-controlled clinical trial was performed from June 2020 to September 2021 in Tabriz, Iran. Patients with DN were recruited from people who were referred to the diabetes clinic of Imam Reza hospital as well as public announcement. Diabetic patients aged between 40 and 70 years, diagnosed with DN, with proteinuria more than > 0.3 g/24 h, and BMI over 27 kg/m^2^and under 40 kg/m^2^ were eligible to participate in the study. Having renal disorders, liver disease, cardiovascular disease, hypo- or hyperthyroidism, cancer, uncontrolled diabetes (HbA1c more than 8 mg/dL), consumers of anticoagulants and non-steroidal anti-inflammatory drugs, alcohol consumption, smoking, pregnancy or breast-feeding, following unusual diets in the last 6 months or consuming energy less than 800 kcal or more than 4200 kcal per day, routine consumption of okra in the last three months, and allergy to okra were considered exclusion criteria of the study. The estimated sample size for the present two-arm parallel study was 25 subjects for each group with a power of 80% and α = 0.05 to investigate a difference of 25 mg/dl in the mean value of fasting blood glucose with a pooled standard deviation obtained from the study of Saatchi et al.^22^

###  Study procedure 

 At baseline, the procedure of the study was explained for all eligible participants and written informed consent was obtained from all subjects. Patients were stratified by gender and randomly allocated either to the intervention (n = 32) or placebo (n = 32) group by block randomization method with block sizes of four (using StataCorp. 2017. Stata Statistical Software: Release 15. College Station, TX: StataCorp LLC). The intervention group received either one 125-mg capsule of DOE (containing 80 mg DOE, 4% Avicel, and 1% magnesium stearate) or carboxymethyl cellulose (CMC) (containing 87 mg CMC) for 10 weeks. DOE and CMC capsules were similar in terms of shape, size, color, taste, and smell. The subjects and investigators were blinded until the end of the study and analysis of data, as the bottles of the supplements were coded with A or B before the study. Moreover, all patients in each group received a list of DN dietary recommendations. Every week, the investigators followed the participants by phone calls to ensure their supplement consumption and check for any possible side effects. The patients were given one bottle of the supplement every 24 days and requested to return their bottle in the next visit to check out the number of remaining capsules and degree of compliance with the supplement. Individuals with less than 90%-compliance were excluded from the final analysis.

###  Assessment of dietary intake, physical activity, appetite, and blood pressure

 Dietary intake was assessed at the onsetof the study and the end of 10 weeks, using a 3-day (2 usual days and one weekend) food record. The patients were trained how to record their dietary intake. The investigators interviewed the patients to check out their dietary records, according to the household measures for determination of the exact portion size. Afterward, the portion sizes were converted to grams and analyzed for energy and nutrient content, using Nutritionist IV software. The physical activity of the subjects were evaluated at the beginning and endpoint of the trial by the International Physical Activity Questionnaire short form (IPAQ-SF).^24^ The physical activity level of the patients was classified as low, moderate, and high.^25^ At baseline and end of the study, a visual analogue scale was also completed to measure the appetite sensation of the patients.^26^ Systolic blood pressure (SBP) and diastolic blood pressure (DBP) of the patients were measured twice in a seated position after ten minutes of rest, using a standard mercury sphygmomanometer. The average of the two measurements was entered to the final analysis.

###  Measurement of anthropometric indices and body composition 

 The weight of the patients was measured while they were wearing light clothes, with 100-g precision, using a Seca digital scale (Seca, Hamburg, Germany). The height was measured barefoot to the nearest 0.1 cm, using a meter reader attached to the wall. Body mass index (BMI) was calculated by dividing weight (kg) by the square of height (m^2^). In addition, waist circumference (WC) was determined by a non-elastic meter with 0.5 cm precision in the narrowest area between the rib cage and the umbilicus. Furthermore, hip circumference (HC), the largest circumference between the waist and knees, was measured by an inelastic meter with 0.5 cm precision. Waist to hip ratio (WHR) was calculated by the followed formula: WC/HC. Body composition indices were assessed using bioelectric impedance analysis (BIA) (MC-780; Tanita, Amsterdam, The Netherlands).

###  Statistical analysis 

 All statistical analyses were performed using IBM SPSS Statistics software (IBM SPSS Statistics, Armonk, USA, version 23). Statistical analysis was conducted with per-protocol approach.^27^ The Kolmogorov–Smirnov test, along with graphical methods, was used to test the normality assumption of data. The data were reported as means ± standard deviation^28^ and median (25^th^, 75^th^) for normally and non-normally distributed data, respectively. Also, qualitative variables were presented as frequency (percent). To check for between-group differences for quantitative data, independent samples *t* test or Mann-Whitney U test was applied, as appropriate. However, for qualitative variables, Fisher’s exact test or Sign test was performed. Paired samples *t* test and Wilcoxon signed-rank test were also used to evaluate intra-group differences, as appropriate. Analysis of covariance (ANCOVA) and quantile regression models (model 1: adjusted for baseline values, model 2: adjusted for baseline values, age, changes in energy intake, BMI, and physical activity) were conducted to control the effect of confounding factors for normally and non-normally distributed data, respectively. *P* values less than 0.05 were set as statistically significant level.

## Results

**Figure 1:**
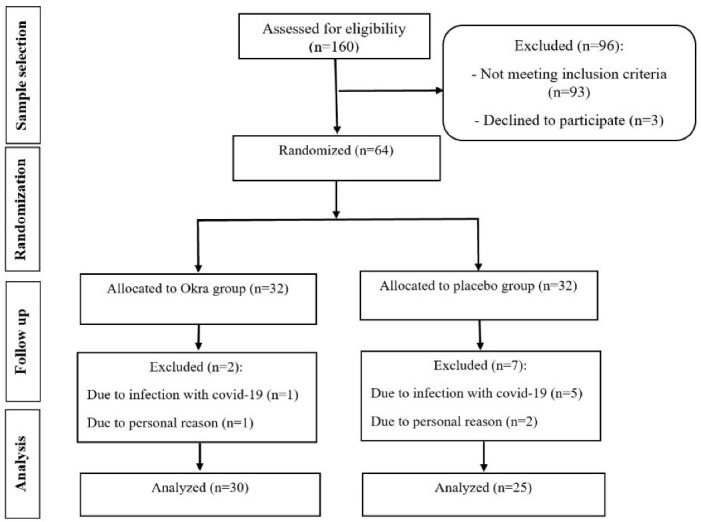
Flow diagram of the study participants

 As shown in Figure 1, out of 64 patients at the baseline enrolled in the study, 55 subjects completed the trial. Seven participants in the placebo group withdrew from the study due to infection with COVID-19 or personal reasons. Also, two patients were excluded from DOE group, due to infection with COVID-19. At the beginning of the study, there was no significant difference between the two groups in terms of demographic variables, including gender, marital status, job, education level, drug use, and duration of diabetes (Table 1). The mean age of the patients in DOE and placebo groups was 62 (SD 7) years and 61.6 (SD 8.5) years, respectively. The mean duration of diabetes in DOE and placebo groups was 16.63 (SD 8.15) years and 17.24 (SD 7.54) years, respectively.

**Table 1:** Demographic characteristics of the study patients

**Variable**	**DOE (n=30) No. (%)**	**Placebo (n=25) No. (%)**	***P*** ** value**
Age (y)	62 ± 7	61.6 ± 8.5	0.864^#^
Gender			0.556^*^
Male	10 (33.3)	6 (24.0)	
Female	20 (66.7)	19 (76.0)	
Marital status			0.684^*^
Single	0 (0)	1 (4.0)	
Married	27 (90.0)	22 (88.0)	
Widow	3 (10.0)	2 (8.0)	
Job			0.196^*^
Unemployed	13 (43.3)	8 (32.0)	
Self-employed	14 (46.7)	11 (44.0)	
Employee	3 (10.0)	6 (24.0)	
Education			0.697^*^
Lower diploma	19 (63.3)	14 (56.0)	
Diploma	8 (26.7)	8 (32.0)	
Bachelors and higher	3 (10.0)	3 (12.0)	
Drugs			0.690^*^
Oral hypoglycemic drugs	14 (46.6)	13 (52.0)	
Insulin	16 (53.3)	12 (48.0)	
Duration of diabetes (y)	16.63 ± 8.15	17.24 ± 7.54	0.777^#^

DOE: dried okra extract. ^#^ Independent sample *t *test. ^*^ Fisher’s exact test.

 The dietary intake and physical activity of the participants were presented in Table 2. According to the results of within-group analysis, energy intake (*P* = 0.047) and carbohydrate consumption (*P* = 0.038) were decreased at the end of the trial only in DOE group, compared to the baseline levels. However, there were no significant differences in the intake of protein and fiber in neither groups. Between-group analyses revealed no significant changes in dietary intake, even after controlling the effects of confounders (baseline values, age, changes in energy intake, and physical activity). Although the level of physical activity was reduced in DOE (*P* = 0.004) at the end of the study, no significant between-group was observed.

**Table 2:** Dietary intake and physical activity of the study patients

**Variable**	**DOE**	**Placebo**	***P*** ** value** ^#^	**Mean change (95% CI)**	***P*** ** value**
Energy (kcal)					
Baseline	1615.99 ± 458.10	1533.36 ± 455.12	0.595	-82.63 (-395.05, 229.78)	0.766^a^
End of trial	1401.43 ± 326.39	1413.32 ± 519.73	0.930	11.88 (-295.91, 283.69)	0.922^b^
Mean change (95 % CI)	- 214.56 (-425.87, -3.25)	- 120.03 (-333.84, 93.76)			
*P* value^*^	0.047	0.245			
Carbohydrate (g)					
Baseline	278.08 ± 93.79	239.99 ± 84.12	0.222	-38.09 (-100.18, 23.99)	0.294^a^
End of trial	234.00 ± 61.56	248.50 ± 94.15	0.562	14.50 (-35.67, 64.69)	0.041^b^
Mean change (95 % CI)	- 44.08 (-85.64, -2.53)	8.51 (-30.78, 47.81)			
*P* value^*^	0.038	0.645			
Protein (g)					
Baseline	57.59 ± 18.54	56.98 ± 22.40	0.928	-0.6 (-14.16, 12.95)	0.496^a^
End of trial	53.75 ± 14.37	50.36 ± 17.08	0.515	-3.38 (-13.82, 7.05)	0.054^b^
Mean change (95 % CI)	-3.83 (-10.94, 3.27)	-6.61 (-18.32, 5.08)			
*P* value^*^	0.278	0.242			
Fat (g)					
Baseline	31.62 ± 11.54	26.45 (21.13, 5614)	0.181	7.31 (-3.54, 18.18)	0.389^a^
End of trial	29.96 ± 12.05	22.18 (19.68, 31.44)	0.531	-2.68 (-11.27, 5.90)	0.206^b^
Mean change (95 % CI)	-1.66 (-8.53, 5.03)	-4.46 (16.14%)			
*P* value^*^	0.615	0.069			
Fiber (g)					
Baseline	15.81 ± 6.13	14.17 ± 5.64	0.424	-1.63 (-5.72, 2.45)	0.789^a^
End of trial	13.68 ± 4.25	13.58 ± 5.48	0.948	-0.10 (-3.30, 3.09)	0.571^b^
Mean change (95 % CI)	-2.21 (-4.75, 0.51)	-0.59 (-3.06, 1.87)			
*P* value^*^	0.110	0.612			
Physical activity (Mets)					
Baseline	609.75 (30, 2009.62)	840 (268.50, 2206.50)	0.611	508.18 (-4156.13, 5172.50)	0.846^c^
End of trial	430.80 (0. 847.12)	462 (198, 1571.25)	0.467	2488.90 (-2544.99, 7522.96)	0.638^d^
Median (% change)	-90.75 (-45%)	0 (-0.07%)			
*P* value^*^	0.004	0.543			

DOE: dried okra extract, BMI: body mass index, Mets, metabolic equivalents ^*^Paired *t* test. ^#^Independent sample *t *test. ^a^ ANCOVA, adjusted for baseline. ^b^ ANCOVA adjusted for baseline, age, changes in energy intake and physical activity. ^c^ Quantile regression, adjusted for baseline. ^d^ Quantile regression, adjusted for baseline, age, changes in energy intake and BMI.

 Anthropometric variables including weight, BMI, WC, HC, and WHR of both groups are illustrated in Table 3. Within-group analysis showed that there was not any significant difference between baseline and end of the trial in terms of weight, BMI, WC, and HC in both groups; however, WHR was significantly reduced in the placebo group at the end of the trial, in comparison to the baseline. Between-group comparisons did not indicate any significant difference for anthropometric variables between DOE and placebo groups. Even after adjusting for the confounders (baseline values, age, changes in energy intake and physical activity), there were no significant effects of DOE supplements on weight, BMI, WC, HC, and WHR.

**Table 3:** Anthropometric indices, BMR and body composition indices of the study patients

**Variabl**e	**DOE**	**Placebo**	***P*** ** value**	**Mean change (95% CI)**	***P*** ** value**
Weight (kg)					
Baseline	80.73 ± 14.02	75.42 ± 10.80	0.129	-5.31 (-12.24, 1.60)	0.764^a^
End of trial	80.52 ± 13.77	75.13 ± 10.86	0.121	-5.38 (-12.24, 1.46)	0.249^b*^
Mean change (95% CI)	-0.21 (-1.06, 0.63)	-0.284 (-1.39, 0.83)			
*P* value^*^	0.610	0.604			
BMI (kg/m^2^)					
Baseline	30.35 ± 5.05	28.64 ± 3.17	0.147	-1.71 (-4.05, 0.62)	0.764^a^
End of trial	30.16 ± 4.89	28.49 ± 2.91	0.139	-1.67 (-3.91, 0.56)	0.557^b*^
Mean change (95% CI)	-0.19 (-0.58, 0.20)	-0.152 (-0.56, 0.26)			
*P* value^*^	0.330	0.456			
WC (cm)					
Baseline	104.32 ± 12.52	102.02 ± 7.06	0.397	-2.29 (-7.95, 3.35)	0.683^a^
End of trial	104.65 ± 11.97	102.15 ± 7.63	0.372	-2.49 (-8.05, 3.06)	0.783^b*^
Mean change (95% CI)	0.32 (-0.77, 1.42)	0.132 (-1.05, 1.31)			
*P* value^*^	0.548	0.820			
HC (cm)					
Baseline	103.44 ± 8.90	99.90 ± 5.17	0.078	-3.53 (-7.66, 0.59)	0.966^a^
End of trial	104.03 ± 8.53	100.93 ± 4.75	0.103	-3.09 (-7.02, 0.82)	0.766^b*^
Median change (%change)	0.00 (1%)	0.00 (1.96%)			
*P* value^*^	0.082	0.167			
WHR					
Baseline	0.99 ± 0.074	± 0.048	0.261	0.01 (-0.01, 0.05)	0.147^a^
End of trial	0.99 ± 0.077	1.00 ± 0.037	0.847	0.001 (-0.03, 0.03)	0.182^b*^
Median change (%change)	-0.005 (-0.09%)	-0.003 (0.50%)			
*P* value^*^	0.861	0.017			
Visceral fat (%)					
Baseline	12.03 ± 4.21	12.00 ± 3.14	0.822	-0.23 (-2.29, 1.83)	0.599^a^
End of trial	12.33 ± 4.23	12.58 ± 3.58	0.819	0.25 (-1.92, 2.42)	0.185^b^
Median (%change)	0.00 (4.34%)	0.00 (8.33%)			
*P* value^*^	0.398	0.162			
Body fat (kg)					
Baseline	26.65 ± 10.07	24.84 ± 8.20	0.558	-1.47 (-6.51, 3.55)	0.636^a^
End of trial	26.82 ± 9.50	25.52 ± 7.53	0.587	-1.30 (-6.07, 3.47)	0.269^b^
Mean change (95% CI)	0.17 (-0.57, 0.92)	0.67 (-0.66, 2.02)			
*P* value^*^	0.632	0.307			
Fat mass (kg)					
Baseline	22.48 ± 10.64	18.34 ± 5.76	0.093	-3.94 (-8.57, 0.67)	0.599^a^
End of trial	22.55 ± 10.08	19.02 ± 5.72	0.120	-3.39 (-7.69, 0.91)	0.314^b^
Mean change (95% CI)	0.06 (-0.61, 0.75)	0.71 (-0.47, 1.90)			
*P* value^*^	0.839	0.224			
Fat-free mass (kg)					
Baseline	59.50 ± 10.53	56.83 ± 10.60	0.285	-3.08 (-8.82, 2.64)	0.454^a^
End of trial	59.47 ± 10.36	56.43 ± 10.33	0.288	-3.04 (-8.72, 2.64)	0.275^b^
Mean change (95% CI)	-0.03 (0.79, 0.72)	0.40 (-0.75, 1.55)			
*P* value^*^	0.929	0.482			
Muscle mass (kg)					
Baseline	56.52 ± 10.04	53.98 ± 10.11	0.287	-2.93 (-8.40, 2.53)	0.454^a^
End of trial	56.50 ± 9.88	53.60 ± 9.84	0.290	-2.89 (-8.31, 2.53)	0.275^b^
Mean change (95% CI)	-0.02 (0.75, 0.7)	0.37 (-0.73, 1.48)			
*P* value^*^	0.941	0.487			
Bone mass (kg)					
Baseline	2.97 ± 0.49	2.84 ± 0.49	0.284	-0.14 (-0.41, 0.12)	0.389^a^
End of trial	2.97 ± 0.48	2.82 ± 0.48	0.267	0.14 (-0.41, 0.11)	0.242^b^
Median (% change)	0.00 (0%)	-0.02 (-1.66%)			
*P* value^*^	1.00	0.423			
Skeletal muscle mass (kg)					
Baseline	32.27 ± 7.27	30.98 ± 7.20	0.433	-1.54 (-5.47, 2.38)	0.544^a^
End of trial	32.37 ± 7.09	30.81 ± 7.02	0.423	-1.56 (-5.44, 2.31)	0.553^b^
Mean change (95% CI)	-0.10 (-0.66, 0.87)	-0.17 (1.22, 0.87)			
*P* value^*^	0.786	0.733			
Total body water (kg)					
Baseline	42.52 ± 8.03	40.74 ± 7.99	0.339	-2.09 (-6.44, 2.25)	0.474^a^
End of trial	42.34 ± 7.80	40.28 ± 7.75	0.338	-2.06 (-6.33, 2.21)	0.373^b^
Mean change (95% CI)	-0.18 (-0.88, 0.51)	-0.46 (-1.34, 0.42)			
*P* value^*^	0.595	0.291			
Basal metabolic rate (kJ)					
Baseline	7278.93 ± 1265.27	6909.45 ± 1189.19	0.220	-407.55 (-1080.95, 254.52)	0.474^a^
End of trial	7277.60 ± 1250.90	6870.04 ± 1166.46	0.226	-407.55 (-1074.86, 259.73)	0.314^b^
Mean change (95% CI)	-1.33 (-86.73, 84.07)	-39.41 (-163.26, 84.43)			
*P* value^*^	0.975	0.517			
Muscle trunk (kg)					
Baseline	31.70 ± 4.98	31.01 ± 5.48	0.195	-1.88 (-4.76, 0.99)	0.345^a^
End of trial	31.72 ± 4.88	29.91 ± 5.16	0.169	-1.90 (-4.63, 0.83)	0.586^b^
Mean change (95% CI)	0.12 (-0.43, 0.68)	-0.10 (-0.80, 0.60)			
*P* value^*^	0.654	0.770			
Fat trunk (%)					
Baseline	24.21 ± 9.11	24.60 ± 6.70	0.839	0.45 (-4.01, 4.92)	0.765^a^
End of trial	24.31 ± 8.64	24.94 ± 5.60	0.748	0.64 (-3.37, 4.67)	0.341^b^
Mean change (95% CI)	-0.10 (-1.84, 1.64)	0.34 (-1.26, 1.94)			
*P* value^*^	0.907	0.662			

DOE: dried okra extract ^*^Paired *t* test; ^#^ Independent sample *t* test. ^a^ ANCOVA adjusted for baseline. ^b^ ANCOVA adjusted for baseline, age, changes in energy intake, BMI, and physical activity. ^b*^ ANCOVA, adjusted for baseline, age, changes in energy intake and physical activity.

Table 3 shows changes in the body composition indices. The results of within-group, as well as between-group analyses, revealed no significant effect of DOE on body composition indices. Considering the effects of confounding variables including baseline values, age, changes in energy intake, BMI, and physical activity made no significant changes in the results.

 Appetite scores of the patients in both groups did not significantly differ within groups, except for a desire to eat salty food (*P* = 0.023) in DOE group and feeling to eat (*P* = 0.024) in placebo group, which reduced significantly at the end of the trial, compared to the baseline (Table 4). Comparison of the mean changes between the two groups did not show any significant differences after adjusting for confounder such as baseline values, age, changes in energy intake, and physical activity.

**Table 4:** Appetite scores of the study patients

**Variable**	**DOE**	**Placebo**	***P*** ** value** ^#^	**Mean change**	***P*** ** value**
Hunger					
Baseline	5.43 ± 2.47	4.92 ± 2.19	0.424	-0.51 (-1.79, 0.76)	0.826^a^
End of trial	4.90 ± 2.17	4.48 ± 2.02	0.465	-0.42 (-1.56, 0.72)	0.886^b^
Median (%change)	- 1.0 (0%)	0 (0%)			
*P* value^*^	0.129	0.134			
Fullness					
Baseline	4.83 ± 2.39	5.24 ± 2.16	0.515	0.40 (-0.83, 1.65)	0.331^a^
End of trial	4.86 ± 2.44	5.60 ± 2.08	0.242	0.73 (-0.50, 1.97)	0.279^b^
Median (%change)	0 (0%)	0 (0%)			
*P* value^*^	0.940	0.131			
Feeling to eat					
Baseline	6 (5, 8)	7 (4, 8)	0.638	7 (-14.28%)	0.99^c^
End of trial	5.5 (4, 7.25)	6 (3, 7)	0.462	6 (-8.33%)	0.748^d^
Median (%change)	-0.5 (-8.33%)	0 (-14.28%)			
*P* value^*^	0.184	0.024			
Desire to eat sweet foods					
Baseline	6 (2, 7)	6 (3, 8)			
End of trial	6.5 (2, 8)	6 (3, 8)	0.779	6 (0%)	0.99^c^
Median (%change)	0 (0%)	0 (0%)	0.734	6 (8.33%)	0.567^d^
*P* value^**^	0.922	0.856			
Desire to eat salty foods					
Baseline	2.5 (1.75, 5)	2 (1.5, 5)	0.863	2 (25%)	0.293^c^
End of trial	2 (1, 4)	2 (2, 5)	0.328	2 (0%)	0.751^d^
Median (%change)	0 (-20%)	0 (0%)			
*P* value^**^	0.023	0.776			
Desire to eat fatty foods					
Baseline	5.33 ± 2.46	3.92 ± 1.95	0.074	-1.41 (-2.63, 0.19)	0.782^a^
End of trial	4.96 ± 2.52	3.88 ± 2.31	0.105	-1.08 (-2.40, 0.23)	0.495^b^
Median (%change)	0 (0%)	0 (0%)			
*P* value^*^	0.233	0.885			

DOE: dried okra extract. ^*^Paired *t *test analysis. ^**^ Wilcoxon signed-rank test. ^#^ Independent sample *t *test. ^a^ ANCOVA adjusted for baseline. ^b^ ANCOVA adjusted for baseline, age, energy intake, physical activity. ^c^ Quantile regression, adjusted for baseline. ^d^ Quantile regression, adjusted for baseline, age, changes in energy intake and physical activity.

## Discussion

 To the best of our knowledge, the present study is the first clinical trial that investigated the effects of DOE on anthropometric variables, body composition, dietary intake, and appetite score in DN patients. Based on the results of this study, although the administration of DOE supplements for 10 weeks significantly reduced energy intake and carbohydrate consumption, it had no significant effect on the whole dietary parameters compared to the placebo. The supplementation also could not significantly affect anthropometric measures, body composition, and appetite score in DN patients.

 Obesity, especially visceral obesity, could develop insulin resistance, dyslipidemia, activation of the sympathetic nervous system, activation of renin-angiotensin-aldosterone system, and kidney compression in the body, all of which increase the tubular reabsorption of NaCl and arterial hypertension. This is followed by albuminuria and kidney injury, which are underlying causes for DN.^29,30^ Our results about the effects of DOE on anthropometric indices are in accordance with the results of Moradi et al^21^ study, who reported that the supplementation of 10 g okra powder for 8 weeks could not improve body weight and BMI in patients with T2DM. Another clinical trial demonstrated that a test meal which contained okra could not affect body weight in overweight subjects with impaired glucose tolerance.^31^ An *in vivo* study showed that feeding okra extract to the obese rats for two weeks did not have any notable effect on body weight.^19^

 In contrast, previous *in vivo* studies indicated the beneficial effect of okra on body weight, such as the study by Fanet al in which the administration of okra polysaccharides (OP) to mice fed a high-fat diet (HFD) could significantly reduce their body weight in comparison with those receiving only HFD.^15^ It was also reported that supplementation with okra powder for 12 weeks inhibited weight gain caused by HFD.^32^ One *in vivo* study mentioned the effects of OP on decreasing the size of white adipose tissue (WAT) and the cells of WAT.^15^

 Although the results about the okra on body weight and body fat are inconsistent, some possible mechanisms have been proposed in previous studies. First, okra is reported to be a rich source of fibers that can delay gastric emptying and cause a full feeling.^33^ Second, bioactive polysaccharides of okra and flavonoids involved in weight management via the regulation of nuclear transcription factors such as peroxisome proliferator-activated receptor-γ and liver X receptors.^34^ It was documented that weight loss needs the reduction of dietary intake as well as the increase in energy expenditure.^35^ Based on our results, although the energy intake of the patients in DOE group was significantly decreased, level of their physical activity was also reduced, in parallel, which could affect the results. Reduced levels of physical activity in patients may be due to the pandemic condition of the coronavirus in which people were encouraged to be quarantined.

 According to the present clinical trial, okra had a significant effect neither on appetite score nor on food intake, compared to the placebo group. In line with our results, Fanet alreported that okra extract supplementation for 2 weeks did not affect dietary intake.^19^ Another *in vivo* study indicated that supplementation with okra seed oil at 400 and 800 mg/kg for eight weeks did not have any effect on food intake of mice.^36^

 The primary mechanism of okra on satiety is related to the fibers of okra which reduced the speed of gastric emptying and caused full feeling, although beneficial effects of okra on the regulation of adipokines such as leptin and adiponectin have also been reported in one study.^37^ The supplement used in the present study did not have any effect on satiety, it is not known whether the intervention was not of adequate dosage or the extraction procedure may reduced the amount of some ingredients such as fibers. Or, the supplement in the form of extract may really have no impact on appetite.

 Given that the finding of the present study decision about the effect of okra on weight and body composition still need further studies on the human sample at different circumstances, the obtined contradictory results against other studies may be due to the differences observed in the methodology and design of different studies; since many of the studies indicated the beneficial effect of okra are *in vitro* and conducted on animal models. The bioavailability of okra in humans may differ from that in animals due to several factors, including intestinal absorption, intestinal microflora metabolism, hepatic and intestinal metabolism, cell uptake, and urinary and biliary excretion that can affect its efficiency. In addition, nearly all of the previous studies have been carried out on diabetes patients with milder disease conditions than those with DN state. In addition, inflammatory status is higher in DN due to increased oxidative stress,^38^ and this makes the comparison of our results with previous okra studies on diabetes difficult. Furthermore, the use of different types of okra products (i.e., powder or extract), as well as different preparation methods of supplements, cultivation area, cultivation condition, and harvest time, can all affect the results.

###  Strengths and limitations of the study

 The present clinical trial had several strengths. To best of our knowledge, this was the first clinical trial which examined the effects of okra extract on dietary intake, anthropometric measures, body composition, and appetite score in patients with DN. Second, all of the participants in both groups received a list of DN-related nutritional recommendations. Third, constant follow-up of the patients was performed by phone calls. However, there were some limitations, as below. First, the dietary intake of the patients seems to be underestimated due to self-reporting nature of food records. Second, the duration of the supplementation was a bit shorter due to budget deficit. It appears that better findings may have been achieved by longer durations of supplementation. However, a long-term study may increase the rate of drop-out of patients.

## Conclusion

 The present study for the first time demonstrated that though supplementation of DOE for ten weeks resulted in decreased energy and carbohydrate intake as well as reduced tendency to salty food in DOE group, it did not have any significant effect on anthropometric variables, body composition, dietary intake, and appetite score, compared to the placebo. However, long-term trials with higher doses of okra are encouraged in future studies.

## Acknowledgments

 The authors would like to thank all of the patients who agreed to participate voluntarily in this study, as well as the research team who collected the data. This research is based on the data obtained from the Ph.D. dissertation of Omid Nikpayam entitled ”The Effect of Okra Hydroalcoholic Extract supplemetation on Glycemic Control, Kidney Function, PPAR-α, PPARγ, TGF-β and Nrf-2 in Patients with Diabetic Nephropathy: A Triple-blinded Placebo-Controlled Clinical Trial” submitted to Tabriz University of Medical Sciences.

## Authors’ contributions

 MSA, ON conceptualized the study. Data collection was carried out by ON, NB, ES, VS, and MSA analyzed the data. The first draft was prepared by ON, with subsequent reviews and revisions completed by MSA. All authors reviewed the final draft and gave the approval to publish.

## Funding

 This work was financially supported by the ‘Research Vice-Chancellor’ of Tabriz University of Medical Sciences (TBZMED), Tabriz, Iran, Grant agreement no:64859.

## Ethical approval

 The protocol of the present study was approved by the Ethics Committee of Tabriz University of Medical Sciences (IR.TBZMED.REC.1399.466) and registered at the Iranian registry of clinical trials (IRCT20110530006652N3).

## Competing interests

 The authors report no declarations of interest.
